# Modeling fine-grained spatio-temporal pollution maps with low-cost sensors

**DOI:** 10.1038/s41612-022-00293-z

**Published:** 2022-10-12

**Authors:** Shiva R. Iyer, Ananth Balashankar, William H. Aeberhard, Sujoy Bhattacharyya, Giuditta Rusconi, Lejo Jose, Nita Soans, Anant Sudarshan, Rohini Pande, Lakshminarayanan Subramanian

**Affiliations:** 1 Department of Computer Science, New York University, New York, NY USA; 2 Swiss Data Science Center, ETH Zurich, Zurich, Switzerland; 3 Columbia University, New York, NY USA; 4 Evidence for Policy Design (EPoD) at the Institute for Financial Management and Research (IFMR), New Delhi, New Delhi India; 5 State Secretariat for Education, Research and Innovation (SERI), Bern, Switzerland; 6 Kai Air Monitoring Pvt Ltd, Gautam Buddha Nagar, UP India; 7 Department of Economics, University of Chicago, Chicago, IL USA; 8 Department of Economics, Yale University, New Haven, CT USA

**Keywords:** Environmental impact, Engineering, Statistics

## Abstract

The use of air quality monitoring networks to inform urban policies is critical especially where urban populations are exposed to unprecedented levels of air pollution. High costs, however, limit city governments’ ability to deploy reference grade air quality monitors at scale; for instance, only 33 reference grade monitors are available for the entire territory of Delhi, India, spanning 1500 sq km with 15 million residents. In this paper, we describe a high-precision spatio-temporal prediction model that can be used to derive fine-grained pollution maps. We utilize two years of data from a low-cost monitoring network of 28 custom-designed low-cost portable air quality sensors covering a dense region of Delhi. The model uses a combination of message-passing recurrent neural networks combined with conventional spatio-temporal geostatistics models to achieve high predictive accuracy in the face of high data variability and intermittent data availability from low-cost sensors (due to sensor faults, network, and power issues). Using data from reference grade monitors for validation, our spatio-temporal pollution model can make predictions within 1-hour time-windows at 9.4, 10.5, and 9.6% Mean Absolute Percentage Error (MAPE) over our low-cost monitors, reference grade monitors, and the combined monitoring network respectively. These accurate fine-grained pollution sensing maps provide a way forward to build citizen-driven low-cost monitoring systems that detect hazardous urban air quality at fine-grained granularities.

## Introduction

Pollution prediction in cities with dense populations can be critical for generating fine-grained policy recommendations and public health warnings^1–3^. The scale of accurate sensor-based monitoring required to achieve this can come at a huge cost and thus inhibit building a dense fine-grained pollution sensing map. The deployment of low-cost particulate matter sensors to replace or augment reference grade pollution air quality monitoring systems has been studied extensively recently, and have addressed issues of calibration^4–6^, design^7,8^, data selection^9^, and personal exposure quantification^10,11^. However, building a highly accurate large scale fine-grained pollution sensing and monitoring map that leverages the size of a pollution network has been largely unexplored. Specifically, modeling the behavior of noisy low-cost sensors in cities with high pollution and population density has not been studied previously, with recent state-of-the-art mapping approaches providing errors only in the range of 30–40%^12,13^. This high error lends the pollution sensing map unusable for policymaking and air quality hazard detection. Prior work on deploying low-cost sensor networks for air pollution have been successful on a small scale (within 2 km radius) with high rates of agreement for PM 2.5 measurements in Southeastern United States^14^. Survey studies have shown that there is a necessity for a paradigm shift towards crowd-funded sensor networks to enable fine-grained sensing-based applications on a large scale^15^. The question of calibration issues in such large scale settings has been explored recently with promising results without the need for significant recalibration^16^ after well-controlled laboratory calibration^17^. PM 2.5 prediction models recently have explored deep neural networks like long-short term memory (LSTM), convolution neural networks (CNN), attention-based models; vector regression, partial differential equations, but focus on a single unified model at a single location, rather than in a large scale sensor network setting^18–24^.

Recent work has also explored the use of distributed sensor networks to gather information on air pollution and other meteorological variables in urban contexts^25–29^. Clements et al. ^30^ provide a comprehensive review of many such works. Researchers have sought to learn more about how pollution sensing systems of low-cost sensors may be deployed in urban contexts^14,31–36^. With the exception of Gao et al. ^36^, who examine the performance of fine particulate sensors in Xi’an in China, most of these deployments have occurred in areas with significantly lower air pollution than the city of Delhi in India. Gao et al. ^36^ also point out that low-cost PM_2.5_ sensors may perform worse in very low pollution environments, suggesting that they may be relatively more useful when particulate concentrations are high. Related approaches in this space can be broadly classified into three groups—spatial interpolation approaches, land-use regression, and dispersion models Xie et al. ^37^, Jerrett et al. ^38^. In the case of dispersion models, they assume that an appropriate chemical transport model is identified along with their parameter values, and a high-quality emissions inventory. In the case of land-use regression models, having access to environmental characteristics that significantly influence pollution is critical. This additional data is often suited for longer range predictions, as the geographical and meteorological data vary over a longer temporal and coarser spatial grids^39,40^.

In this paper, we describe a methodology to model and predict urban air quality at a fine-grained level using dense and noisy, *low-cost sensors*. There are two main questions we seek to answer in this paper—(i) how can we use a network of low-cost and portable air quality monitors in order to build a fine-grained pollution heatmap in a city that provides accurate prediction?, (ii) does it help to augment existing monitoring networks by the local governments with low-cost air quality sensors?

We deploy a network of 28 low-cost sensors, many of them concentrated in the south Delhi area, in collaboration with Kaiterra^41^, a company that makes low-cost air quality monitors and air filters. We dramatically increase the density of the deployment by 28× in Delhi (area 573 *mi*^2^) with 28 sensors, compared to previous deployments (Xi’an - area 3898 *mi*^2^, 8 low-cost sensors). Further, the large longitudinal dataset we have been able to capture over 2 years as compared to prior work, which captured at most a few weeks of data, allows us to model long-term seasonal changes and train more complex neural network models that can adapt to seasonal and daily patterns. We build on prior work and model the pollution network in its entirety, with prediction models at each sensor location using data from near-by sensor locations.

We model pollution at any location in Delhi as measured by the concentration of fine particulate matter (PM_2.5_) measured in μgm^−3^ using historical data of up to 8 h from all the sensors in the network. We make this choice of building a fine-grained pollution sensing map over shorter timelines to leverage the primary advantage of low-cost sensors while overcoming the drawback of noise by aggregating numerous spatio-temporal measurements. By learning the variability of each of these noisy measurements through message passing neural networks (MPRNN) which have the ability to model each sensor separately, we learn to not only separate the signal from the noise, but build an accurate sensing network of low-cost sensors that achieves <10% root mean squared earror (RMSE) in predicting up to one hour in advance over a fine-grained spatio-temporal grid as compared to baseline modeling approaches that provide 30% RMSE. By using a sparse network of sensors, whose signals are shared through neural network embeddings, we learn to capture the information from nearby sources that might affect the readings of nearby sources (e.g., factory) and ignore the ones which are heavily localized (e.g., food cart). Such an accurate, fine-grained pollution sensing map (≤10% MAPE) is usable by policymakers in deciding which neighborhoods of the city need interventions to improve the air quality and population health. To the best of our knowledge, we are the first in attempting to model a city-scale sensor network deployment with low-cost sensors augmenting high-quality government monitoring stations. With a sensor network the size of a city, with 60 sensors spread across the city of Delhi (700 sq km), capturing spatio-temporal variations and constructing accurate pollution maps necessitates modeling each sensor separately. By increasing the scale and addressing the corresponding modeling challenges, our work has widespread implications for pollution sensing and its low-cost deployability.

## Results

Our data consists of PM_2.5_ concentration data averaged to the hour from the 28 low-cost sensors and the 32 government monitors, a total of 60 monitors, collected over a period of 24 months, from May 1, 2018, to May 1, 2020. We use the until Oct 30, 2019 for training (75%) and hold out the remaining (25%) for testing. We report two criteria—the RMSE and the mean absolute percentage error (MAPE). We evaluate our models on the data from the combined set of our 28 low-cost sensors and the 32 government monitors, as well as separately on each set. For each of these locations, we compare our model-based predictions with the ground truth of the measurement of the pollution sensor.

**Fig. 1:**
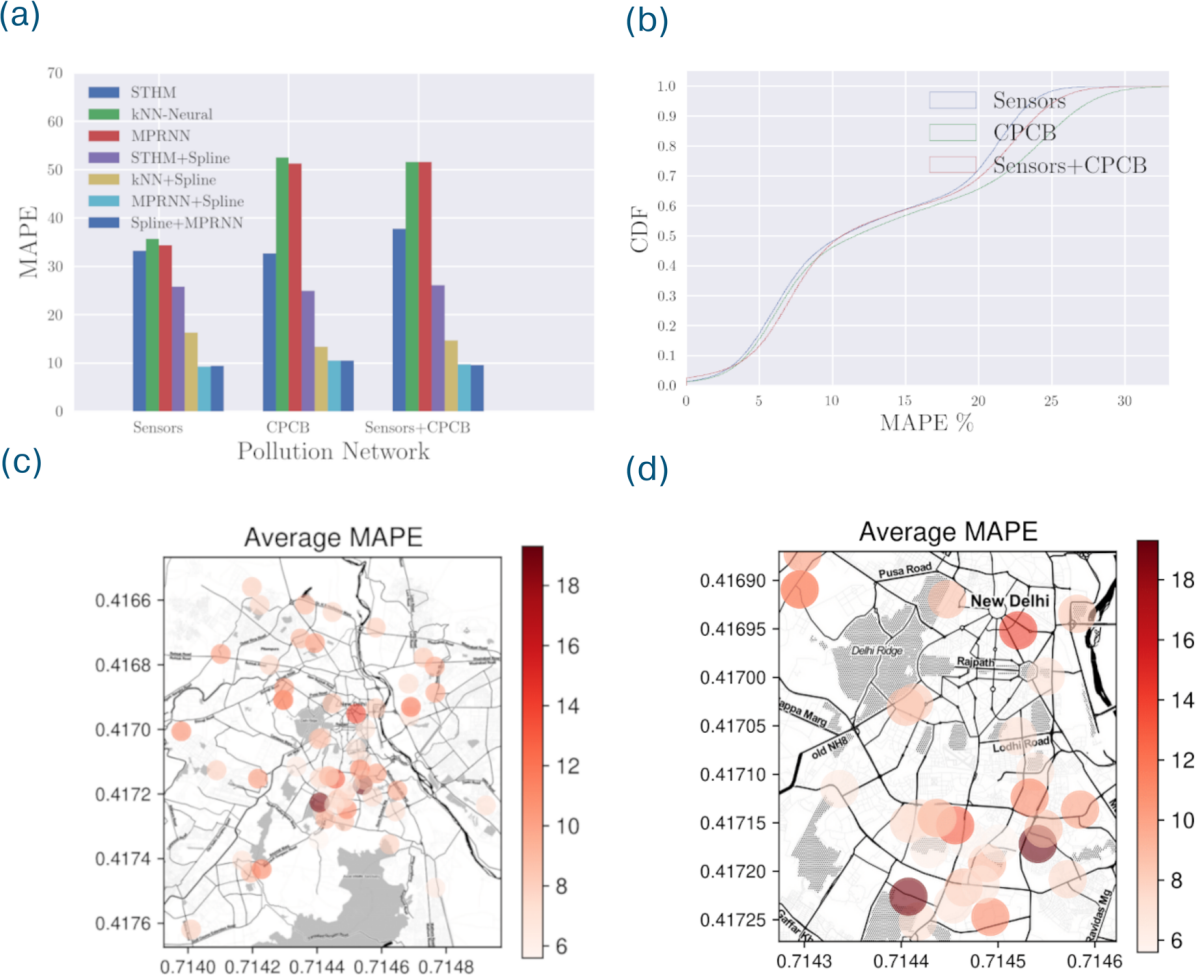
Prediction errors of PM_2.5_ during the test period (Nov 1, 2019–May 1, 2020) shown as the mean absolute percentage error (MAPE) of the ground truth and predicted PM_2.5_ concentration. In this period, the PM_2.5_ concentration values ranges between 0 and 1000 μgm^−3^, and average value being ~130 μgm^−3^. **a** Bar plot comparing our methodology with other competing approaches. We note that modeling spatiotemporal interactions using a neural network such as MPRNN and accounting for intra-day periodic patterns in the form of spline corrections together make a big difference in the performance. **b** Distribution of MAPE for the best performing model - Per-Sensor Spline with STHM imputation + MPRNN, across all the locations shown as a cumulative density function (CDF). **c** Prediction errors of the best performing model (MPRNN+Spline) at every monitoring location on the map. **d** Errors of the final prediction zoomed into the regions with highest concentration of sensors (New Delhi and South Delhi).

Overall, the MPRNN model with imputed data using STHM along with the spline correction provides a very highly accurate estimation of the PM concentration level across all locations (ref Table 1). The best performing model is able to predict PM_2.5_ concentrations with an average RMSE of 10.1 μgm^−3^ and MAPE of 9.6% across all the locations and over the testing period. While estimating a spline per location provides the best predictive performance, we note that using an average spline across all observed locations only marginally increases the RMSE and MAPE errors. The average spline is computed after averaging the data over all the locations. Across all locations, the median RMSE and MAPE are 9.15 μgm^−3^ and 8.64% respectively (ref Fig. 1). The best case values are 4.28 μgm^−3^ and 5.57% respectively, and the worst case values are 24.1 μgm^−3^ and 19.64% respectively. The location where we have minimum MAPE is at a location in Green Park, a very busy area of south Delhi, further validating the need for fine-grained pollution sensing in a large city like Delhi.

**Table 1:** RMSE and MAPE of prediction of PM concentrations, averaged across all the sensor locations.

Model	Our sensors		Govt monitors		Combined	
	*RMSE*	*MAPE*	*RMSE*	*MAPE*	*RMSE*	*MAPE*
STHM	29.5	33.2%	38.3	32.7%	31.4	37.8%
k-NN neural network	38.8	35.7%	69.7	52.6%	54.2	51.6%
MPRNN	37.1	34.4%	65.2	51.3%	56.3	51.6%
Per-sensor spline	25.1	32.8%	60.4	49.1%	47.3	36.5%
STHM + spline	21.8	25.8%	27.2	24.9%	24.2	26.2%
k-NN neural network + per-sensor residual spline	11.6	16.3%	18.1	13.4%	12.8	14.7%
MPRNN + per-sensor residual spline	9.8	10.2%	13.2	11.7%	10.4	12.6%
Per-sensor spline + Residual MPRNN	10.1	10.5%	14.7	12.2%	10.7	13.5%
Per-sensor spline with STHM imputation + MPRNN	9.5	9.4%	12.6	10.5%	10.1	9.6%
MPRNN with STHM imputation + average residual spline	10.1	9.8%	13.2	10.9%	11.2	10.3%

The RMSE is in units of μg/m^3^. The best performing model is shown in boldface. The Per-sensor spline with STHM imputation followed by the use of MPRNN to estimate residual errors performs the best and has significantly lower RMSE and MAPE than any of the models that do not combine these steps. Using just a cubic spline or STHM or MPRNN in isolation results in a significant increase in the RMSE and MAPE errors. Replacing the per-sensor spline with an average spline does not significantly affect the RMSE and MAPE errors. The STHM model is primarily useful in filling in missing values and only provides a minor improvement to the MPRNN + per-sensor spline model. Another baseline method where we replace the MPRNN with k-Nearest Neighbors increases the MAPE and RMSE errors.

### Spatial variations

**Fig. 2:**
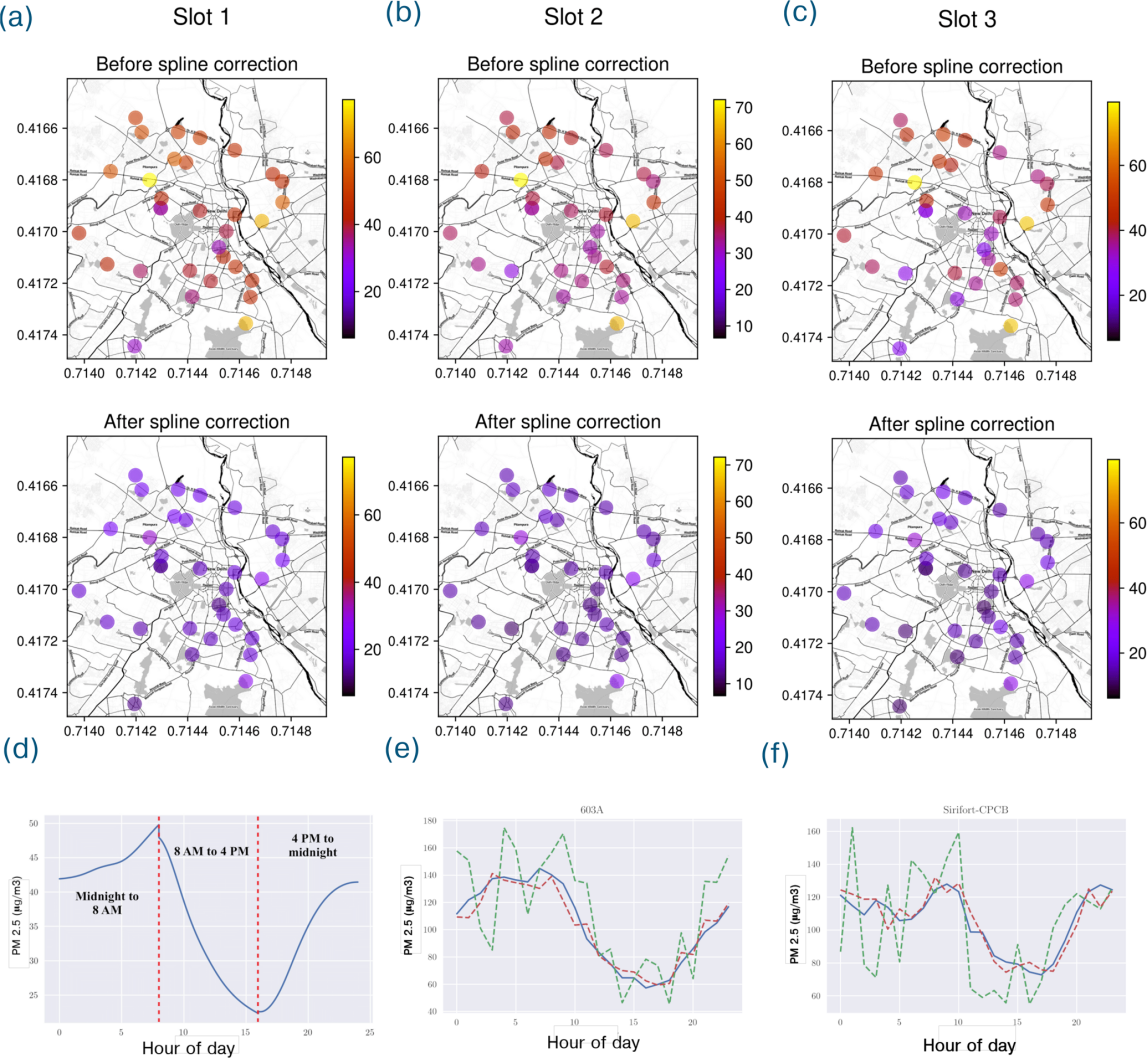
The interpretation of the spline correction, and its effect on the residual. The top two rows show the distribution of the residuals (in PM units of μg/m^3^) over space, before and after the spline correction. Three different splines were fitted over the residuals in three different time slots in the day. We observe that for the most part, locations that exhibited high residual errors after MPRNN fit (in the upper quantiles of the residual error distribution) continued to show high error (relative to other locations) even after spline correction, even though the magnitude of the residual does decrease. This phenomenon is partially explained by the high baseline values of the sensors with high residual errors, that is often coupled with high variance in measurement. **a** Slot 1 (12 AM–8 AM). **b** Slot 2 (8 AM–4 PM). **c** Slot 3 (4 PM–12 AM). **d** Composite cubic spline correction consisting of three splines fitted for three non-overlapping parts of the day—midnight to early morning (12 AM to 8 AM), midday (8 AM to 4 PM), and evening to midnight (4 PM to 12 AM). **e** Ground truth PM_2.5_ (blue), along with MPRNN prediction (green) and final prediction after spline correction (red) at one of our sensor locations in Chanakyapuri in New Delhi. **f** Ground truth PM_2.5_ (blue), along with MPRNN prediction (green) and final prediction after spline correction (red) at the CPCB monitor at Sirifort in South Delhi.

The 3-way cubic spline fit shows a common trend of baseline pollution rising steadily up to 8 am, then decreasing up to 4 pm and then increasing again until midnight. We note that this is the composite polynomial model of the PM concentrations in an average day (ref Fig. 2). The median error of this model is about 40 μgm^−3^ at each of the three windows, 12 am–8 am, 8 am–4 pm and 4 pm–12 am, and this is reduced to about 10 μgm^−3^ post the neural network model fit on the residuals. Figure 2 and Supplementary Fig. 2 show the per-sensor splines and the average spline in detail. Not only do the per-sensor splines vary widely across space, we notice that regions with significantly high spline residual errors like the sensors A838, E8E4, and 2E9C in Supplementary Fig. 2, are all located in central locations of Delhi with well established commercial activity like Connaught Place, Sardarjung Enclave and Lado Sarai respectively. Further, in Supplementary Fig. 2, the outliers with significantly high residual error splines among the government monitoring stations are Patparganj DPCC, Punjabi Bagh DPCC, and DKSSR DPCC. While Patparganj is situated next to an industrial area, Punjabi Bagh is a well-known residential locality with established commercial activity centers, and DKSSR, short for Dr. Karni Singh Shooting Range, is a shooting range located in the outskirts of Delhi next to an interstate highway. The diversity of these splines across various geographical regions further indicate the need to model fine-grained pollution profiles in seemingly remote as well as central locations of Delhi. We also note that the average spline can sufficiently operate for bootstrapping at locations where we do not have enough sensor data to begin with.

For the most part, locations that exhibited high residual errors after MPRNN fit continued to show high error (relative to other locations) even after spline correction, even though the magnitude of the residual decreases. This phenomenon is partially explained by the high baseline values of the sensors with high residual errors, that is often coupled with high variance in measurement.

### Effect of network size and training data

The fewer the monitors we used in our hybrid model, the greater was the final prediction performance. As Supplementary Fig. 3 shows, with only one monitor in the network, the predictive errors are about 35 and 20 μgm^−3^, respectively, for the low-cost sensor network and government network. However, as we include data from more nodes in the network, final prediction error drops sharply to about 15% and then gradually tails off at about 10%. The error flattens out about 30 sensors, which is approximately the number of sensors of each type that we have in our experiment. We infer that having an even denser deployment likely adds little value to the predictive performance. Further, decreasing the amount of training data to train the model shows that at minimum, one year of data is required to capture the seasonal trends and achieve RMSE of almost 10% (Supplementary Table 3).

## Discussion

The low MAPE and RMSE across all monitors in Delhi provided by our Per-Sensor Spline+MPRNN with STHM imputation model are significant as it means that our model can detect hazardous air quality with high precision. The RMSE error is significantly lower than the observed variance in PM_2.5_ concentrations in a day, making it useful for short-term and intraday analyses as well. The WHO air quality standards prescribe that PM_2.5_ levels should not exceed 5 and 15 μgm^−3^ at an annual and daily average levels, while the Indian Government air quality standards prescribe 40 and 60 μgm^−3^, respectively. We note that for the 60 sensors, Delhi has exceeded these prescribed levels 371 out of the 641 days on a daily level, across 2 years of our measurement. The 9.6 % MAPE error that we are able to achieve, corresponds to the ability to detect hazardous air quality as per Indian government standards with 93.5% precision and 90.8% recall. This further indicates that the low error rate we have obtained leads to an almost exact prediction of hazardous air quality. This enables citizen-driven sensing where pollution sensor readings can be crowdsourced, and effective policy interventions like clean energy policies that penalize construction sites that have PM_2.5_ levels more than 25% higher than the nearest monitoring center can be operationalized^42^. Specifically, the improvement in predictive power is achieved in specific pollution hotspots like bus stations, markets, etc. (Fig. 1). In addition, we can provide transparency of the overall average pollution of the city^43^ and contribute towards increasing the co-benefits of clean energy policies^44,45^.

### Calibration

Since the data used to measure the model performance is new, it is important to understand the spatial variations and heterogeneity in measurements that underlies the sensor network. To further ensure that the improvement in model’s prediction performance is better than the noise in the data, we performed extensive calibration of the sensors. For this, we leveraged the calibration performed in-house by the sensor manufacturer (Kaiterra^46^) (more info in Appendix) which confirms that re-calibration is not required^47^, and also perform validation by comparing our sensor readings with the readings provided by the nearest government pollution monitoring station. Supplementary Figure 5 shows the cross-calibration of the average pollution value reported by the 28 government monitors with the average value of the 18 sensors in our testbed in the locality of South Delhi. We observe that the sensors have been fairly well calibrated with the reference monitors and report a similar average value across the city despite individual sensor level and spatio-temporal variations. This provides confidence in the data generated from this pilot to be useful as a reference for pollution modeling and forecasting.

Further, we also performed a nearest neighbor calibration where we compute temporal correlation of our sensor with the nearest government monitoring station of that sensor. Supplementary Table 4 shows that on average the correlation coefficients are >0.8, which indicates that there is no statistical significant difference between them on average (t-test, confidence level: 0.05, *p*-value: 0.0011). Further, in Supplementary Fig. 4, we see that when we order our sensors by the nearest neighboring government station, the cross-correlations between our sensors are correspondingly aligned, with high correlation between nearby sensors and low correlation between farther sensors. This further emphasizes the importance of the improvement in modeling as it significantly improves the prediction capabilities of a fine-grained sensor network, which can capture spatial variations in pollution of Delhi.

The development of fine-grained pollution sensing maps at low-costs can further catalyze the deployment of such monitoring networks in other polluted cities, where the pollution networks are sparse. With citizens procuring, deploying, and modeling pollution of cities accurately, this paper provides a way forward for developing high-quality fine-grained pollution sensing maps.

## Methods

### Summary

We model the spatio-temporal prediction problem as a graph prediction problem, where we predict a value at every node at a certain time using as input the historical values from neighboring nodes. In our setting, each sensor location *v* ∈ *V* is a node in an undirected graph. Assuming that air pollutants diffuse uniformly in all directions and exert their influence throughout our region of interest, in this case the greater Delhi region, we make the graph complete, where an edge exists between every pair of nodes. The end goal is to train a model that predicts at any node, the pollution level, measured in terms of the concentration of fine particulate matter PM2.5, at time *t* given one or more readings from neighboring locations prior to *t*. The first step is to interpolate the gaps in the data. We use a geostatistics model for this task, called the Spatio-temporal Hierarchical Model (STHM). Then we fit a cubic spline based on daily trends at each sensor location, and then finally train a Message-Passing Recurrent Neural Network (MPRNN) (Section 4.4) to predict residuals over the baseline. In order to account for the amount of influence based on the pairwise distances, we include the Euclidean distance between sensors as part of our feature embedding in our message-passing formulation. We test this model by predicting values at locations where sensors, and therefore ground truth information, are present, but the model is generalized enough to be used to predict at locations where there is no ground truth data available. If $y_{v,t}$ is the reading of the sensor at location *v*, at timestamp *t*, and ${\hat{y}}_{v,t}$ is our corresponding prediction, the prediction model aims to minimize the mean absolute percentage loss:

1 $${\rm{MAPE}} = \sum_v \sum_t \frac{|{\hat y}_{v,t} - y_{v,t}|}{y_{v,t}}$$

Our pollution forecasting model for estimating the PM_2.5_ particulate matter concentration across space and time consists of three important steps. Given the variations in data availability across our pollution sensors, the first step of our method uses a standard Spatio-Temporal Hierarchical Model (STHM) to estimate the missing data. Our STHM model is a standard statistical modeling framework from geostatistics that combines multiple sources of information, accommodates missing values, and computes predictions in both space and time. Based on daily variation patterns observed at each of the pollution sensors, the second step in our method estimates a three-way cubic spline at each sensor location, one for each disjoint 8 h interval in a 24 h period (12 am to 8 am, 8 am to 4 pm and 4 pm to 12 am), representing three different patterns in the PM_2.5_ variations. The cubic splines for each sensor represented a baseline level of PM_2.5_ concentration. The cubic splines may provide a good approximation to the overall average daily variations across sensors but do not capture short term spatio-temporal variations represented by the residual errors in the baseline. The final step of our method is to train a Message-Passing Recurrent Neural Network (MPRNN) across the pollution monitoring points to estimate the residual errors from neighboring sensors. We will briefly describe the characteristics of our data and then explain the cubic spline and MPRNN methodology in this section. We refer the reader to the supplementary text for a detailed description of the STHM model.

### Data

The data used for the modeling the air pollution levels in Delhi was sourced from a combination of 32 local government monitors and a network of 28 low-cost sensors deployed by us in various locations of Delhi from May 2018 to May 2020. The average availability of each of these sensors are about 90 and 30% over the measured period, respectively. This disparity is attributed to a variety of factors such as disconnection for periodic necessary calibration, network outages and periodic servicing of sensors. The sensors are calibrated against the government sensors, by conducting a longitudinal comparison study by measuring in proximity to the location of the government monitoring centers. The locations and their summary statistics of the sensors by location is given by the Supplementary Tables 1 and 2, and are shown visually in the box plots in Supplementary Fig. 1.

### Cubic splines

We observe that on a daily basis, depending on the time of the day and the location, there is a low-frequency component that makes up an approximate “baseline level” of PM concentration. Based on this observation, we fit a piecewise polynomial function, called a spline, to model this low-frequency component. We divided a single day into a number of epochs and fit a spline for each epoch. Prior to implementing the cubic splines, we observed that the residual errors from the MPRNN model exhibits different errors at different times in the day. We then proceeded to fit cubic splines based on the daily spatio-temporal patterns per sensor and per location. For example, if our prediction error follows a temporal pattern of say, higher prediction error in the morning, while lower in the afternoon, we can leverage this fitting separate splines for morning and afternoon to subtract out this component. The spline can be of any order, but given our residual error patterns, but we found that piecewise cubic spline works best. Suppose at time *t* and location *v*, the raw PM value is given by *y*_*v*,*t*_. Then, the piecewise spline to predict *y*, with time period *p* is given by:

2 $${\hat{y}}_{p}(v,t)={\alpha }_{v,p}* {t}^{3}+{\beta }_{v,p}* {t}^{2}+{\kappa }_{v,p}* t+{\nu }_{v,p}$$

Note that the chosen parameters per sensor *α*_*v*,*p*_, *β*_*v*,*p*_, *κ*_*v*,*p*_, *ν*_*v*,*p*_, where *p* ∈ {“morning”, “afternoon”, “evening”}, depend on the patterns in our residual errors and are fit accordingly to minimize the root mean-squared residual error:

3 $${\rm{RMSE}}(v)=\mathop{\sum}\limits_{t}\mathop{\sum}\limits_{p}\sqrt{{(y(v,t)-{\hat{y}}_{p}(v,t))}^{2}}$$

### Message-passing recurrent neural network

MPRNN, based on refs. ^48,49^, is a neural network architecture that is applied on a graph in order to predict values at each node in the graph. This approach enables to us incorporates spatial interactions between each pair of nodes as “messages” that are broadcast from every node to its neighbors. Each node has a modified version of a long short term memory (LSTM) network that iterates between message-passing and the recurrent computations.

Suppose *y*_*v*,*t*_ is a quantity of interest at node *v* and time *t*, for which we would like to build a predictive model. Mathematically, we would like to learn a function ${{{\mathcal{F}}}}$ such that, ${y}_{v,t+1}={{{\mathcal{F}}}}({v}_{1},{y}_{{v}_{1},t},{v}_{2},{y}_{{v}_{2},t},\ldots ;{v}_{j}\in {{{\mathcal{V}}}})$ where the set ${{{\mathcal{V}}}}$ denotes the set of all the nodes in the graph. A recurrent neural network unit is assigned to each node in the graph, with each node *v* maintaining a hidden state *h*_*v*,*t*_ at time *t*. Through a message-passing phase and a time-recurrent phase, our model infers the next hidden state, *h*_*v*,*t*+1_ from which the PM value at *v* is decoded. A message-passing operation allows one segment to observe the hidden state of its neighboring segments.

The computation proceeds in five steps, as five layers of the neural network. In the first phase, the observation phase, the input observations ${Y}_{t}=\{{y}_{v,t}| v\in {{{\mathcal{V}}}}\}$ at time *t* are encoded into *h*_*v*,*t*_ by the observation operation *O*_*v*_. In the second and third phases, one or more iterations of messaging (*M*) and updating (*U*) operations are performed to propagate the observations in the graph. In the fourth phase, for each node, a time-recurrent operator *T*_*v*_ utilizing an LSTM unit takes as input the final hidden state *h*_*v*,*t*_ and predicts the next hidden state *h*_*v*,*t*+1_. The final phase is the readout operation *R*_*v*_, which decodes the hidden state to produce the output value to be predicted ${\hat{y}}_{v,t+1}$. These five steps are shown below. The message function takes as input the hidden states of a pair of nodes *v* and *n* and the Euclidean distance between them, *d*_*v*,*n*_ as the influence of the pollution at a given location on the pollution at another location would depend on the distance between them. Hence, we include the distance in the embedding.

4 $${h}_{v,t}={O}_{v}({h}_{v,t-1},{y}_{v,t})$$

5 $${m}_{v,t}=\mathop{\sum}\limits_{n\in V-v}M({h}_{v,t},{h}_{n,t},{d}_{v,n})$$

6 $${h}_{v,t}=U({h}_{v,t},{m}_{v,t})$$

7 $${h}_{v,t+1}={T}_{v}({h}_{v,t})$$

8 $${\hat{y}}_{v,t+1}={R}_{v}({h}_{v,t+1})$$

For a selection of nodes ${{{\mathcal{W}}}}$ in the graph, the components of the model $\{{O}_{w},M,U,{T}_{w},{R}_{w},| w\in {{{\mathcal{W}}}}\}$ are defined. During inference, the states ${H}_{t}=\{{h}_{w,t}| w\in {{{\mathcal{W}}}}\}$ are maintained at each time step. The hidden state for each segment is initialized at *t* = 0 randomly during training and evaluation ${h}_{v,0} \sim {{{\mathcal{N}}}}(0,1)$.

#### Training and validation

We used the data from May 1, 2018, to Nov 1, 2019, a period of 18 months, as the training period. The number of samples we had for training were 166,979 from our low-cost sensor network, and 371,806 from the government network, resulting in a total of 538,785 samples. The model was trained at each sensor location, using as input data from all the other monitors except itself, over the entire training period. We used the Adam optimizer^50^ with a learning rate of 0.001, and ran the training for 30 epochs to ensure a robust and well-trained model. To validate the model, we used the remaining 6 months data from Nov 1, 2019, to May 1, 2020. The number of ground truth samples available in this period were 20,408 and 91,493 in the low-cost network and government network, respectively, resulting in a total of 111901 samples. However, only 12 out of the 28 low-cost sensors were operational in the testing phase, since many of them had not been serviced properly, partly owing to the COVID-19 pandemic. The testing error reported under Results (§2), therefore, shows the predictions tested at 12 low-cost sensor locations and 32 government monitors, a total of 44 locations combined. Further, to understand the implications of availability of less data during training, we evaluated our model as shown in Supplementary Table 3 and found that with training data less than a year, our model’s performance significantly decreases as seasonal trends are not well captured.

#### Implementation

**Fig. 3:**
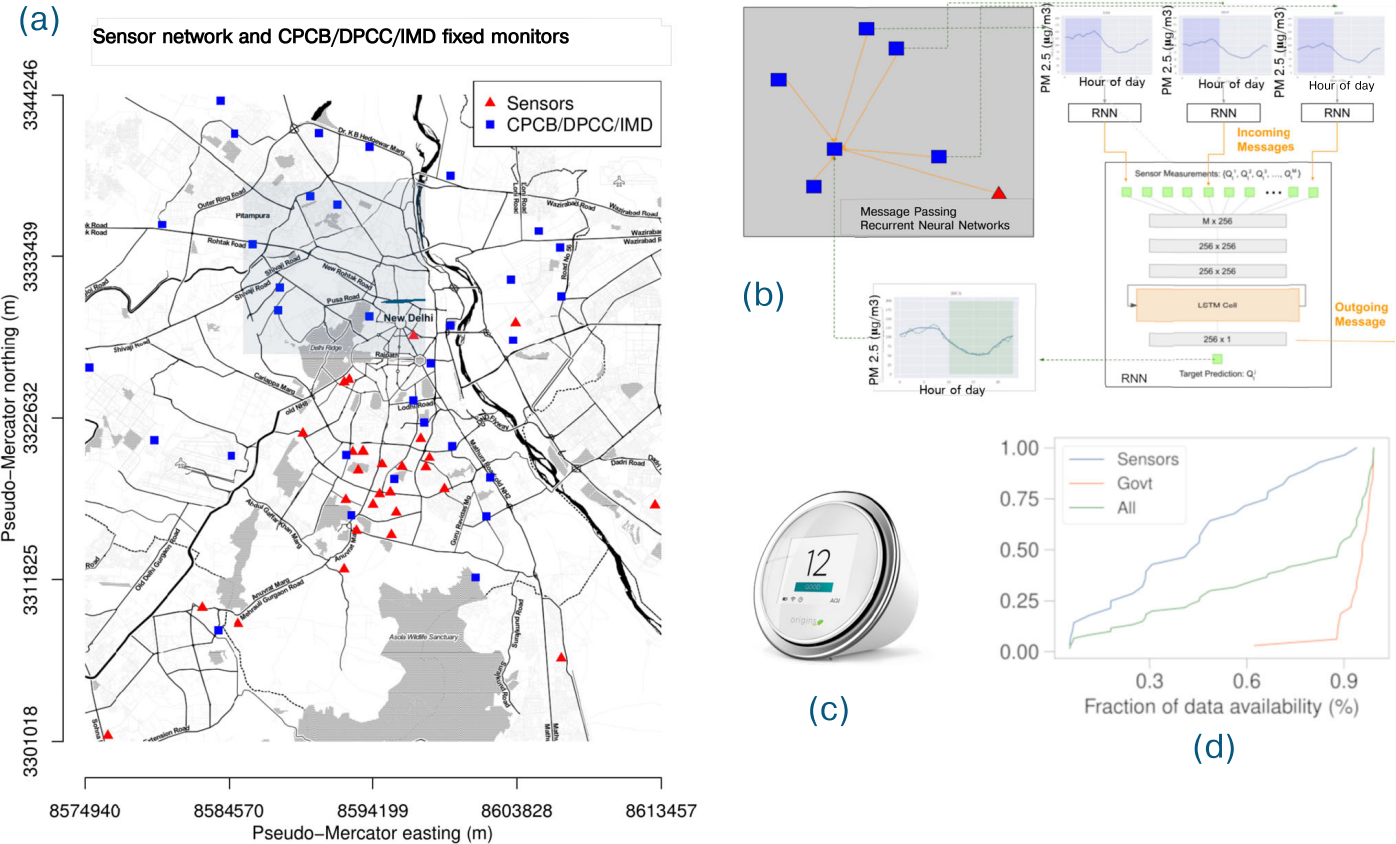
Message passing recurrent neural network for pollution monitoring in Delhi. **a** Network of air quality monitors in the entire greater Delhi region. **b** Model architecture, showing *M* sensor inputs feeding into the layers and producing a single real output, illustrated by zooming on the selected region in (**a**). The computation goes from top to bottom. The green boxes represent input PM concentrations from a set of locations, the gray boxes the hidden linear transformation layers, with the numbers in the boxes representing the number of internal parameters to be learned, and the orange box shows the RNN with the LSTM cells. Here 256 is the embedding size of the hidden layer messages passed, that was chosen empirically based on performance. The final output is the single real value of PM concentration. The input to the RNN is the vector output of length 256 from the hidden layer. More details are in the supplementary text. **c** Sample model of a low-cost sensor. **d** Our experimental testbed of monitors, and the quality of the PM_2.5_ data obtained. We had to contend with frequent outages and communication issues that plagued our sensor network and affected data availability.

The MPRNN is implemented using the *Deep Graph Library*^51^ and PyTorch ^52^ in Python. The model diagram is shown in Fig. 3.

### Baselines

We contrast our combined model with two alternative modeling approaches in order to set a baseline to benchmark the MPRNN model performance. The first one is the STHM itself, a state-of-the-art spatio-temporal modeling methodology. When the STHM is used solely for the prediction, it performs poorly, as it does not model unknown non-linear spatial dependencies due to dispersion. The second baseline is an alternative neural network formulation that collects information from a specified number (*K*) of nearest neighbors to a location *L*, and feeds them into a trained recurrent neural network, to predict the value at *L*. Unlike the MPRNN, this model does not account for explicit spatial influence between every pair of sensors, thus allowing us to see how a more simplified multi-variate non-linear model might perform. We call this model the *k*-Nearest Neighbor (*k*-NN) Spatial Neural Network.

## Supplementary information

Supplementary Material

## Data Availability

The data that supports the findings of this study comprises two parts—the PM2.5 data from the government monitors and the data collected from our low-cost sensor network. The former is public data and can be accessed here^53^. The data can also be provided by the authors upon request. The latter is third-party data and the authors are bound by a confidentiality agreement with Kaiterra, the makers of the low-cost sensors, and can only be made available for confidential peer review, if requested by reviewers, within the terms of the data use agreement and if compliant with ethical and legal requirements.

## Code availability

All the relevant code can be obtained upon request from the corresponding author. The code is also available on GitHub: https://github.com/shivariyer/epod-nyu-delhi-pollution.
