# Supplementary material for: Modeling fine-grained spatio-temporal pollution maps with low-cost sensors
Source: NPJ Clim Atmos Sci. 2022 Oct 12;5(1):76. doi: 10.1038/s41612-022-00293-z (PMC9555706; doi:10.1038/s41612-022-00293-z)
Supplement: Supplementary file 1 — Supplementary Material [file 41612_2022_293_MOESM1_ESM.pdf]

Modeling fine-grained spatio-temporal pollution maps with low-cost sensors

Shiva R. Iyer[a], Ananth Balashankar[a], William H. Aeberhard[b], Sujoy Bhattacharyya[c,g], Giuditta Rusconi[d,g], Lejo Jose[h], Nita Soans[h], Anant Sudarshan[e], Rohini Pande[f], and Lakshminarayanan Subramanian[a]

[a]Department of Computer Science, New York University, New York NY, USA

[b]Swiss Data Science Center, ETH Zurich, Zurich, Switzerland

[c]Columbia University, New York NY, USA

[d]State Secretariat for Education, Research and Innovation (SERI), Bern, Switzerland

[e]Department of Economics, University of Chicago, Chicago IL, USA

[f]Department of Economics, Yale University, New Haven CT, USA

[g]Formerly affiliated with the Evidence for Policy Design (EPoD) at the Institute for Financial Management and Research (IFMR), New Delhi, Delhi, India, at the time of contribution to this work

[h] Kai Air Monitoring Pvt Ltd

Corresponding author email: lakshmi@cs.nyu.edu

August 19, 2022

Supplementary Methods

Calibration at Kaiterra: Each device is calibrated and tested to ensure that it adheres to our technical specifications. Almost all air quality monitors on the market are tested over just two or three levels of pollutant concentration, and only a handful of these data points are used in the calibration process. Kaiterra products, however, use over 6,000 individual measurements during the calibration and verification process. From testing chamber design to performance certification, we created a process that verifies that every sensor is properly calibrated before and after assembly. We have designed a testing environment that goes well beyond the required industry standards. Below, we outline the 3 components of our testing environment.

1. **Testing chamber**: An air-tight testing chamber is used to simultaneously test up to 360 monitors over a set amount of time and in varying conditions. This custom-made testing chamber is 90 cubic meters. Monitors are mounted on angled shelving that has been specifically designed to allow high airflow between the devices, preventing pollutant buildups from forming within the test chamber – the uniformity of the air within this chamber is extremely important. To ensure that each device is tested under consistent conditions, ventilation equipment controls the temperature and amount of the pollutant released.

2. **Careful calibration**: Certain sensors require calibration during the manufacturing process. For these parameters, specific concentrations of pollutants are introduced into the test chamber. This process is carried out remotely outside the test chamber so that no human interference can influence the concentration of pollutants inside. Once the concentration of pollutant has stabilized within the chamber and is uniform throughout, all devices within the chamber will be remotely calibrated to this concentration.

3. **Comprehensive measurement validation**: Every single device that comes off of the production line must be fully tested for particulate matter over several hours, and all readings must fall within specification. While most air quality monitors on the market are tested over two or three different levels of concentration and only a handful of data points are used, the device takes over 6,000 individual measurements during the test process. Each of these mea-

surements are processed onboard and sent to the cloud in real-time. Software in the cloud will process these readings and compare them to measured reference values within the test chamber. The divergence between the monitor's measured readings and the reference readings are calculated for every single data point and verified against the specifications of the device. Only devices that fulfill the published technical specifications will leave the testing chamber. Any failed devices will enter a separate process to determine where the failure occurred, and once identified, to resolve it, before returning to the production line to be fully re-tested.

Localized Cloud Calibration A laser-based detector uses MIE scattering to count particles and determine their size. Based on the changing intensity of light picked up by this sensor, individual particles can be counted and their size determined. These individual particles are placed into groups (known as bins) based upon their size. Particles 0.3-0.5 microns might be placed in one bin, while 0.5-1.0 micron particles would be placed in another bin. Assumptions can be made about the density and refractive index of particles, both of which affect the mass concentration, and this is what most particle counters do. The vast majority of consumer-grade devices simply have a default correction factor built into the device which is always used, regardless of the size of the particles. Professional-grade devices will usually allow users to enter in these details manually, but knowing the density of the particles being measured is no easy feat, and usually requires sending a sample of particles to a lab for analysis. In real life, the density and optical properties of particles will be heavily affected by geography. The particles are extremely different in various geographical areas, and using the wrong factor in calculating mass concentrations will lead to results that can be way off.

Simply using a default factor for all devices means that results will be wrong more often than they are right. Seeing that each city has a different pollution profile, the location will affect the accuracy of the device. Most devices need to be calibrated by hand using data sheets from each city or sent to a professional calibrating service, our device can get all of this data from a central server and make the necessary tweaks automatically. The key to long-term accurate readings is having an algorithm that can take these changing factors into account in real-time.We set up numerous co-locations around the globe with reference grade monitors that provide particulate readings as mass

concentrations (e.g. BAMs and TEOMs). We then analyze the readings of our optical monitors against these devices in real-time. From this data, we are able to identify the key properties necessary to update the algorithm inside our devices. Based on the location selected for a device, the device will be provided with an updated calibration algorithm every five minutes. This means that as the wind blows from one side of a town to the other, and as the makeup of particles in the air changes, the algorithm inside every device based in that town will change accordingly, in real-time.The correlation between the outdoor unit and the reference grade E-BAM was also analyzed as shown in Supplementary Figure 6.

Consistency between sensors: Since the readings from devices may drift apart due to various factors, the sensors were randomly tested for consistency. They were taken from the site at random and the tests were conducted for consistency between them. Two sensors were compared by locating them in the same testing environment and making measurements for 7 days. The 2 sensors produced very similar readings in the comparison. Data was collected every 60 sec for 7 days, resulting in 10,080 readings. The full dataset was plotted for PM2.5 and correlation coefficient was checked. The regression equations showed very good agreement between the sensors for both PM2.5 along with the R^2 in Supplementary Figure 7.

Supplementary Tables

Location	Count	Minimum	Maximum	Median	Mean	Std Dev
Anand Vihar, Delhi - DPCC	18911	0.2	985.0	79.5	122.1	120.0
Ashok Vihar, Delhi - DPCC	21278	0.2	972.0	66.0	105.9	110.6
Aya Nagar, New Delhi - IMD	20368	0.1	954.0	56.3	76.9	74.4
Burari Crossing, New Delhi - IMD	9593	0.1	989.7	85.7	125.8	121.2
CRRI Mathura Road, New Delhi - IMD	21242	0.0	973.8	64.8	97.0	97.7
Dr. Karni Singh Shooting Range, Delhi - DPCC	19908	0.1	967.5	50.0	82.1	87.1
DTU, New Delhi - CPCB	20854	0.5	968.2	67.6	105.9	102.3
Dwarka-Sector 8, Delhi - DPCC	21615	1.0	958.2	61.5	95.7	94.1
IGI Airport (T3), New Delhi - IMD	20640	0.1	867.2	52.9	79.8	79.7
IHBAS, Dilshad Garden,New Delhi - CPCB	20913	1.6	989.6	70.5	95.3	86.6
ITO, New Delhi - CPCB	19804	0.8	989.2	76.0	107.1	94.2
Jawaharlal Nehru Stadium, Delhi - DPCC	21251	0.2	929.0	55.8	90.3	95.0
Jahangirpuri, Delhi - DPCC	21414	0.2	994.0	79.0	119.3	114.3
Lodhi Road, New Delhi - IMD	20248	0.1	980.8	55.8	77.3	72.5
Major Dhyan Chand National Stadium, Delhi - DPCC	21680	0.2	985.8	59.2	86.5	81.3
Mandir Marg, New Delhi - DPCC	20839	0.3	945.0	63.8	90.5	85.0
Mundaka, Delhi - DPCC	19654	0.5	988.5	72.5	116.6	120.2
NSIT Dwarka, New Delhi - CPCB	21601	1.0	997.5	77.9	97.6	76.8
Nehru Nagar, Delhi - DPCC	21692	0.2	997.5	62.8	110.2	121.9
Okhla Phase-2, Delhi - DPCC	21474	1.0	987.0	59.2	94.3	95.6
Patparganj, Delhi - DPCC	21602	0.2	983.0	58.2	89.7	90.0
Punjabi Bagh, Delhi - DPCC	21049	0.0	988.0	63.8	103.0	112.5
Pusa, Delhi - DPCC	19142	0.2	978.0	57.5	91.9	92.6
Pusa, New Delhi - IMD	19770	0.1	986.2	52.7	77.0	77.7
R K Puram, New Delhi - DPCC	19383	0.5	877.2	63.2	92.9	93.6
Rohini, Delhi - DPCC	21310	1.0	967.0	73.0	116.7	115.2
Shadipur, New Delhi - CPCB	20937	1.0	997.2	69.2	97.6	89.3
Sirifort, New Delhi - CPCB	20735	0.2	994.2	61.8	92.8	89.9
Sonia Vihar, Delhi - DPCC	21176	0.8	984.0	64.0	99.4	97.5
Sri Aurobindo Marg, Delhi - DPCC	20116	0.2	992.8	52.5	80.6	80.4
Vivek Vihar, Delhi - DPCC	21344	1.0	957.2	63.0	101.2	104.7
Wazirpur, Delhi - DPCC	21401	1.0	969.8	74.0	118.7	117.7

Supplementary Table 1: **Summary Statistics of Government Pollution Monitors.** The number of readings, minimum, maximum, median, mean and standard deviations of the $PM_{2.5}$ concentrations detected

Location	Count	Minimum	Maximum	Median	Mean	Std Dev
GK-1 Pamposh Enclave	10173	2.2	959.0	66.3	113.7	113.9
Safdarjung Enclave Block B1	3804	1.5	444.7	60.0	85.9	74.5
Anand Niketan	8655	0.9	809.3	55.6	91.4	92.5
Lado Sarai	1733	7.0	617.5	112.0	135.6	99.8
Sarvodaya Enclave	810	1.0	319.0	32.2	45.1	39.8
GK-1 N Block	954	6.5	241.7	55.5	67.4	41.5
Malviya Nagar	9253	3.0	743.4	81.2	125.3	117.0
Panchsheel Park	6959	1.9	664.1	56.4	97.0	100.2
Gurugram Sector 24	2776	6.2	427.1	41.5	52.2	40.4
Malcha Diplomatic Enclave Block C	6274	2.8	1047.4	65.9	103.0	100.1
Faridabad Sector 32	13975	2.8	909.8	81.0	119.1	104.5
Aya Nagar Extension	8003	0.1	1117.4	56.5	102.8	113.7
Chhatapur	2753	9.6	1145.8	81.7	125.6	120.5
Safdarjung Enclave Block B4	6998	1.0	507.8	54.7	89.3	84.0
Fulbright House	5103	4.1	827.7	106.4	145.6	124.7
Sadiq Nagar	20079	0.3	1110.8	53.6	90.7	93.4
Hauz Khas Village	4404	0.0	487.0	50.8	87.6	88.0
Preet Vihar	11223	4.2	1142.9	69.5	114.9	114.5
Saket	859	5.3	371.1	56.3	75.1	61.1
Gurugram Sector 49	10246	1.4	696.8	62.2	97.3	90.5
US Embassy	10952	1.4	915.7	62.7	95.3	87.1
Yusuf Sarai	7220	2.1	563.0	54.7	88.0	86.2
Lajpat Nagar	6959	1.0	507.8	54.9	89.9	84.2
Defence Colony	4721	3.0	481.5	72.8	105.3	92.7
Sarvapriya Vihar	1009	13.0	274.7	63.1	72.6	41.5
Noida Sector 104	15586	0.1	954.8	63.3	99.5	94.4
Safdarjung Enclave Block B4	4538	7.8	1205.5	99.2	143.7	124.6
ISI Delhi	16075	0.3	836.4	54.8	91.0	89.5

Supplementary Table 2: **Summary statistics of low-cost pollution sensor network.** The number of readings, minimum, maximum, median, mean and standard deviations of the $PM_{2.5}$ concentrations detected

Training Data (in months)	Our sensors		Govt monitors		Combined	
	RMSE	*MAPE*	*RMSE*	*MAPE*	*RMSE*	*MAPE*
2	40.3	40.1%	43.8	42.9%	41.6	40.9%
4	36.3	35.1%	38.2	37.2%	36.6	35.4%
6	28.3	27.1%	30.1	29.7%	28.2	27.5%
8	22.1	21.8%	24.5	23.6%	22.5	21.7%
10	18.2	18.1%	21.4	20.5%	19.6	18.5%
12	10.5	10.4%	13.7	12.0%	11.5	10.7%
14	10.2	10.1%	13.4	11.8%	11.2	10.5%
16	9.9	9.8%	13.1	11.3%	10.6	10.2%
18	9.5	9.4%	12.6	10.5%	10.1	9.6%

Supplementary Table 3: **Effect of decreasing the size of training data in our best performing model (STHM+Spline+MPRNN).** This shows more than 15% RMSE if less than one year of data is used for training. Further, seasonal trends are important, as models trained from May - June 2018 (first row), do not generalize to test periods between Oct 2019- May 2020. Hence, longitudinal overlap is required for a model that can generalize across months.

Our sensor	Nearest Government Station	Distance (in m)	Correlation (PM 2.5)
113E	NehruNagar-DPCC	2557	0.8738
1FD7	RKPuram-DPCC	440	0.7483
20CA	RKPuram-DPCC	2824	0.8177
2E9C	SriAurobindoMarg-DPCC	947	0.7837
3ACF	SriAurobindoMarg-DPCC	1425	0.9081
498F	Sirifort-CPCB	1967	0.7692
4BE7	Sirifort-CPCB	1949	0.8821
56C3	Sirifort-CPCB	809	0.9219
5D7A	AyaNagar-IMD	1614	0.3116
603A	MandirMarg-DPCC	3919	0.8713
72CA	DKSSR-DPCC	6882	0.8581
8E2A	AyaNagar-IMD	1260	0.6692
91B8	SriAurobindoMarg-DPCC	3167	0.8734
97D7	RKPuram-DPCC	1020	0.8605
A838	MDCNS-DPCC	1942	0.9135
A9BE	Sirifort-CPCB	846	0.8932
BB4A	RKPuram-DPCC	1175	0.8625
BC46	Patparganj-DPCC	1030	0.9197
BFDC	SriAurobindoMarg-DPCC	2617	0.7599
C0A7	AyaNagar-IMD	8961	0.6261
CBC7	MandirMarg-DPCC	4162	0.8773
D804	Sirifort-CPCB	1123	0.8348
DF07	NehruNagar-DPCC	1497	0.8472
E1F8	JNS-DPCC	964	0.9440
E47A	Sirifort-CPCB	1227	0.5079
E486	CRRIMathuraRoad-IMD	9794	0.8346
E8E4	RKPuram-DPCC	1056	0.8721
EAC8	SriAurobindoMarg-DPCC	962	0.9126

Supplementary Table 4: **Validation of our sensor's readings based on correlation with the nearest government sensor.** This indicates that while there is individual variation among our sensors, the high correlation coefficient indicates that there is neigborhood based correlation between our sensors.

Supplementary Figures

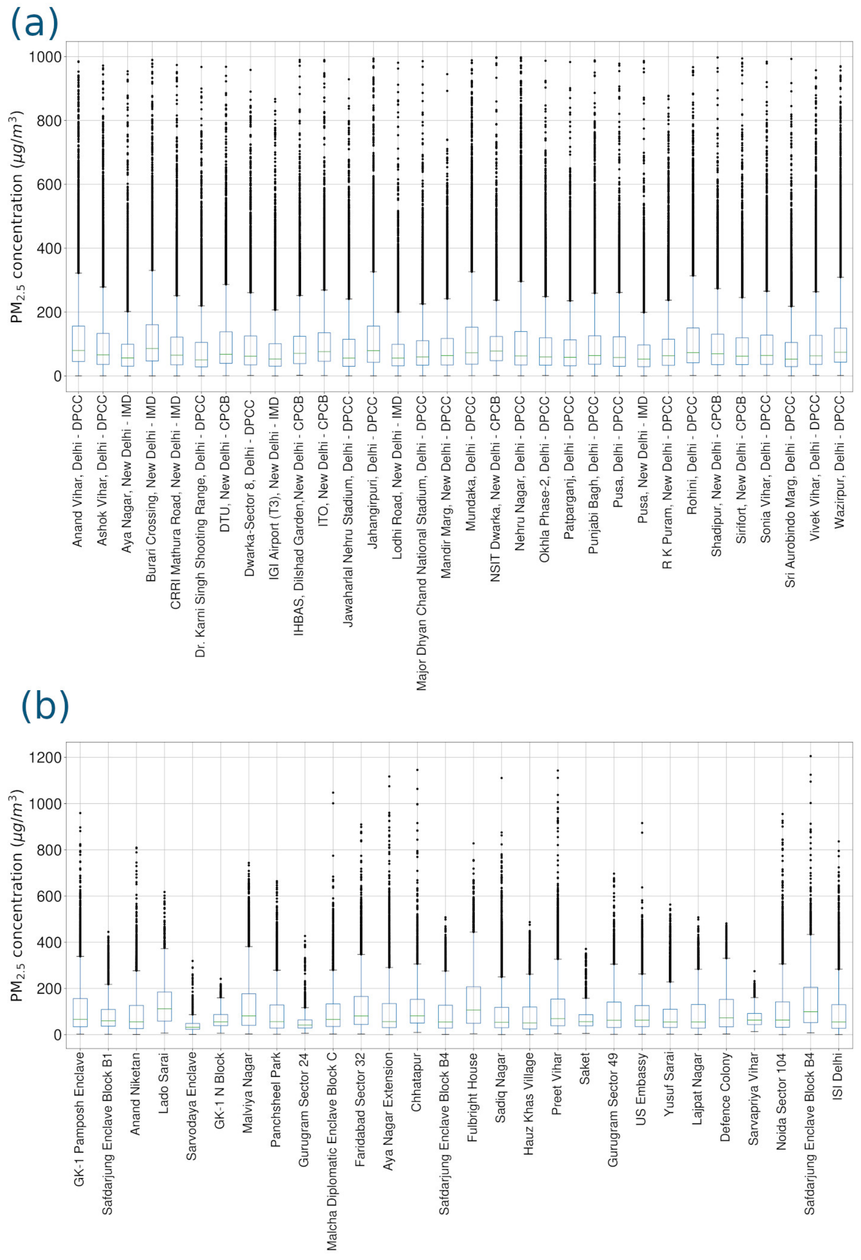

Supplementary Figure 1: **Boxplots of the distribution of PM$_{2.5}$ concentration from the monitors in (a) government network and (b) our low-cost network.** The center-line indicates the median, the bounds of the box indicate the 25th and 75th percentile values, the whiskers indicate the range within which 99.3% of the data exists, while the rest (0.7%) of the bubbles are outliers.

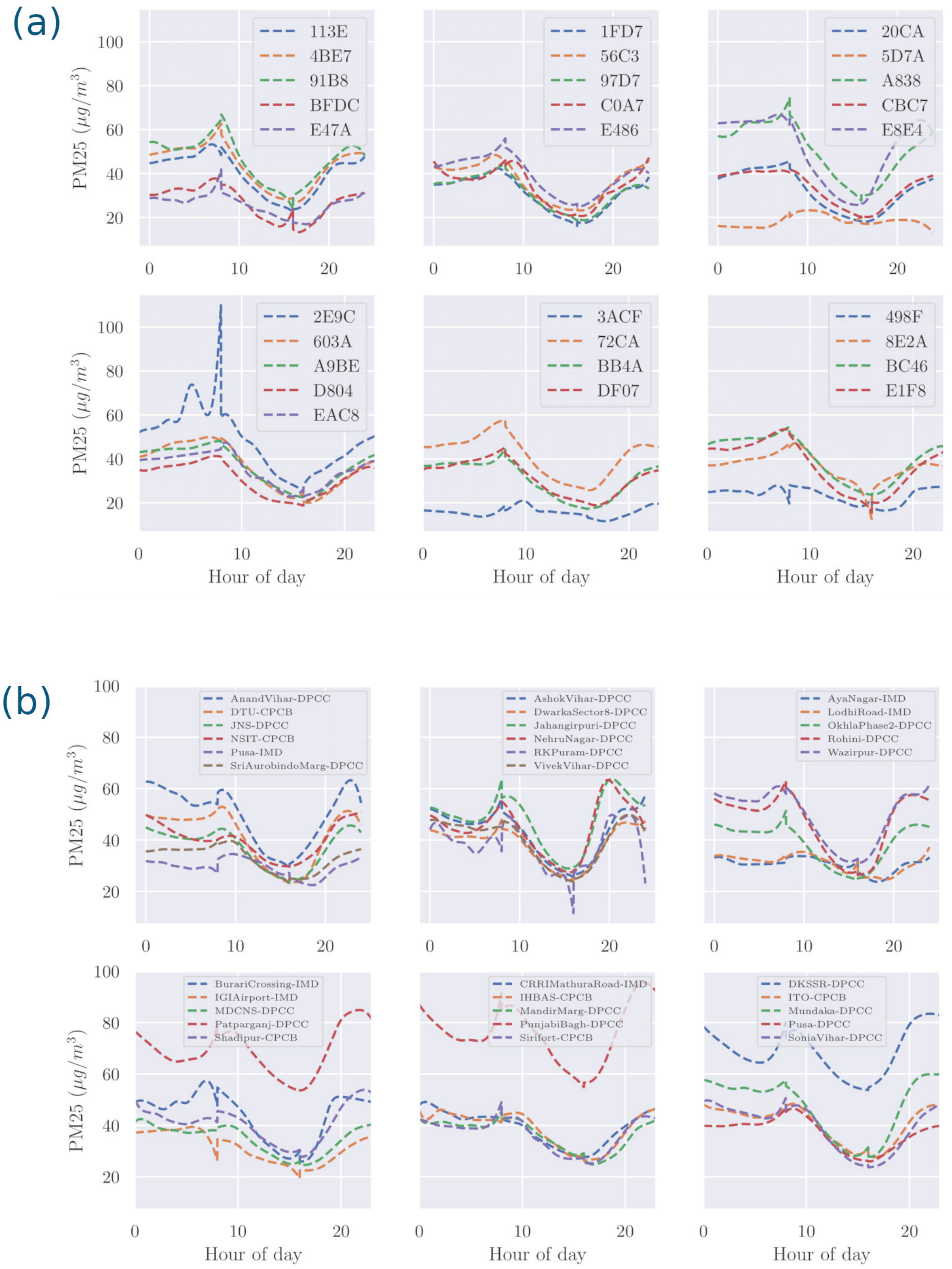

Supplementary Figure 2: **The daily variations in the splines learned for each of the sensors.** This shows that there are temporal patterns which when incorporated into a prediction model can significantly improve prediction accuracy. Each color shows a different sensor location. Each plot shows about 4-5 sensor locations for the sake of readability. The first six plots show the low-cost sensor locations, and the next six show the government monitors. **(a)** Splines for each of the 28 low-cost sensors (4 digit sensor IDs are shown for brevity rather than the full location names) **(b)** Splines for each of the government pollution monitors

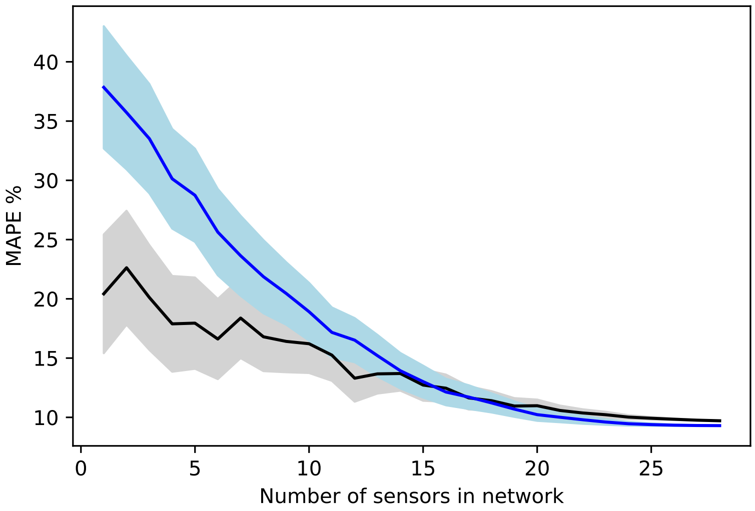

Supplementary Figure 3: **The impact of sensor network size on prediction error.** The blue line shows the errors for our low-cost sensors, and the black for the government monitors. We see that the more sensors we use in our model, the better the performance of the model in terms of the prediction error. The error flattens out about 30 sensors, which is approximately the number of sensors of each type that we have in our experiment. We infer that having an even denser deployment likely adds little value to the predictive performance.

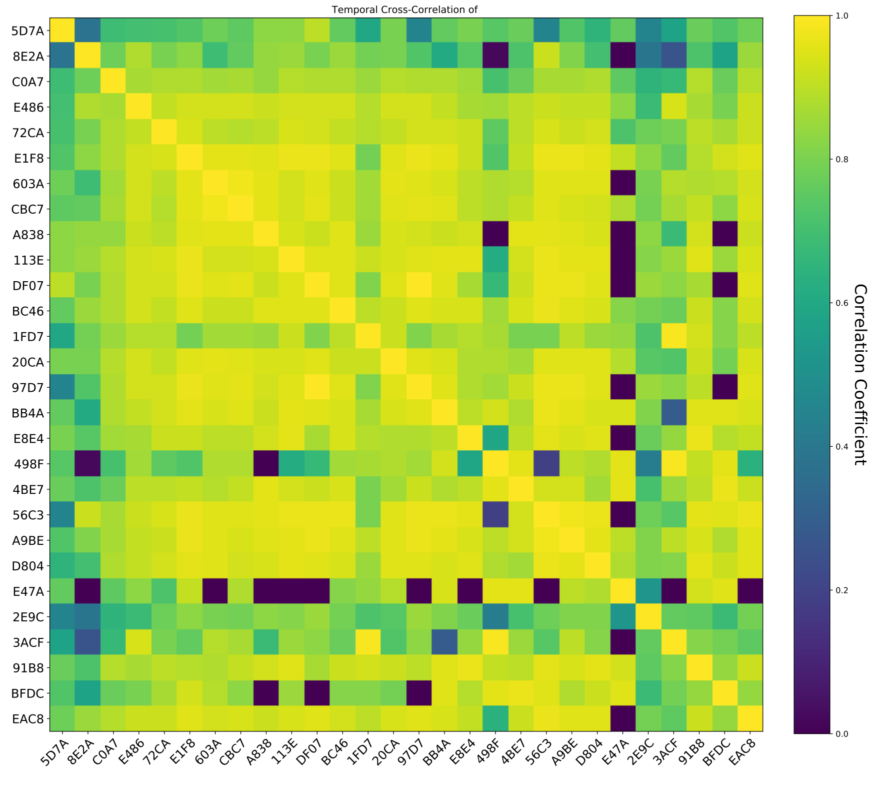

Supplementary Figure 4: **Temporal Cross-correlation heatmap between our sensors grouped and ordered by the corresponding nearest government sensor in Delhi.** This further shows that on average, correlation in nearby sensors (closer to diagonal) are higher than ones that are further away (away from diagonal), thereby showing spatial variability in pollution in Delhi.

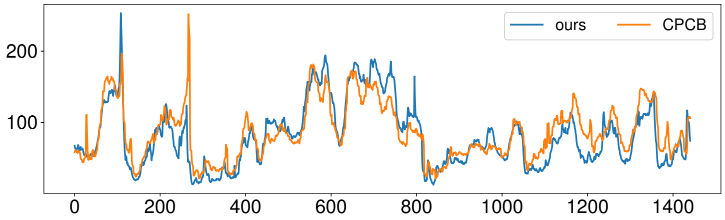

Supplementary Figure 5: **Time series of average readings of our 18 sensors along with the average of the 28 local government monitors in the locality of South Delhi.** This shows that on-average our sensor networks are highly correlated, while providing capability for sensing fine-grained variations.

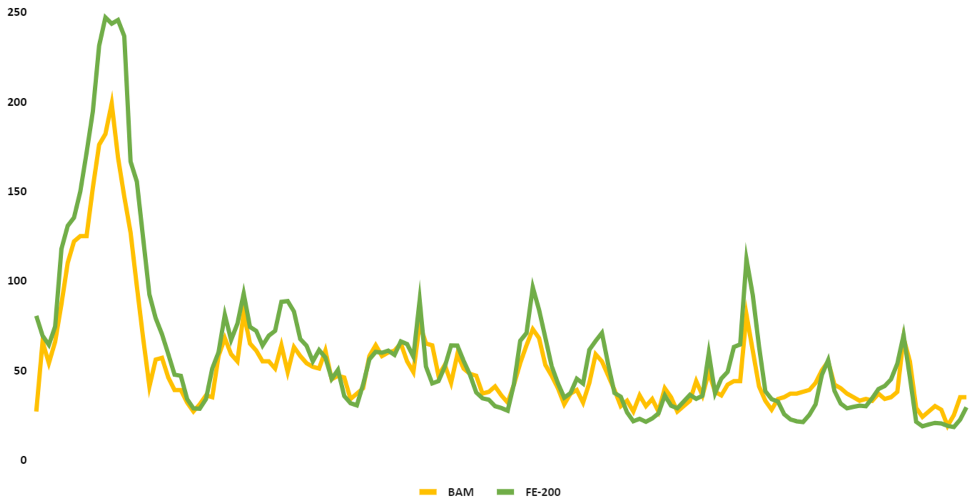

Supplementary Figure 6: **Correlation with calibration sensor for the period of Feb 2019 to April 2019.** The data is represented in one-hour averages for the entire duration and the R2 was 0.96 for the said period.

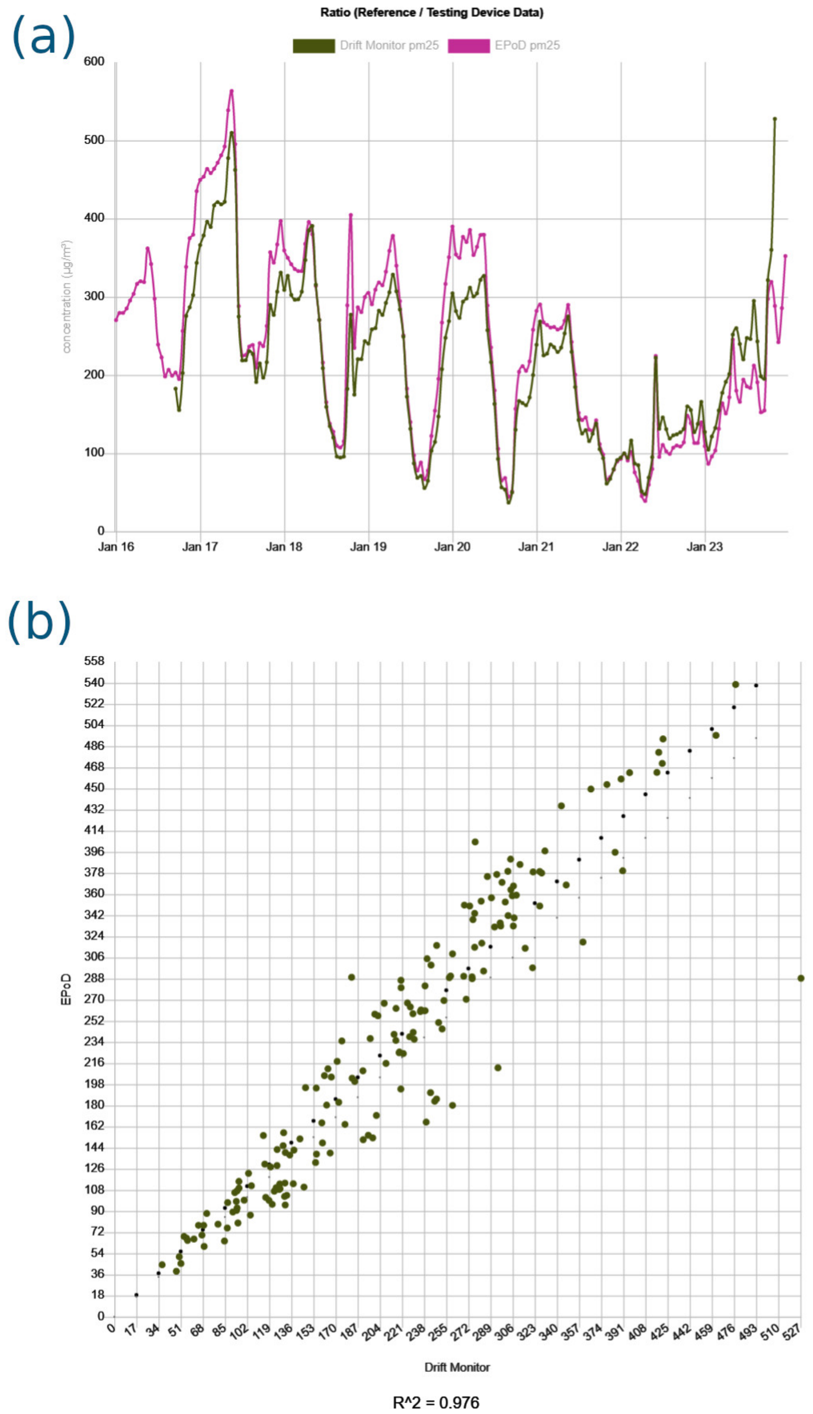

Supplementary Figure 7: **Temporal correlation for sensor calibration near EPoD, New Delhi.** The calibration results shows that as compared to reference drift monitor, our sensor near EPoD, New Delhi has a high temporal correlation with $R^2 = 0.976$.
